# The orbitalauricular chord of *Alligator*: The unusual functional linkage between the earflap and eyelid of Crocodylians

**DOI:** 10.1111/joa.13752

**Published:** 2022-08-24

**Authors:** Bruce A. Young, Bryson Grondel, Peace Preston, Michael Cramberg

**Affiliations:** ^1^ Department of Anatomy Kirksville College of Osteopathic Medicine Kirksville Missouri USA

**Keywords:** blinking, collagen, connective tissue, submergence

## Abstract

One of the distinctive features of the Crocodylia is the presence of a superficial meatal chamber the aperture of which is regulated by two earflaps. The movements of the upper earflap have been detailed by multiple workers, however, the mechanics of the lower earflap remain unresolved. The present study was undertaken to document the mechanics of the lower earflap in the American alligator, *Alligator mississippiensis*, and to explore the functional bases of coordinated movements between the lower earflap and lower eyelid in this species. This anatomical system was examined using a combination of fresh dissection, histology, and micro‐CT analyses applied to post‐embryonic specimens. The rostral margin of the lower earflap is tightly bound to a block of dense connective tissue herein termed the orbitalauricular chord. The orbitalauricular chord is anatomically distinct from both a ligament and a tendon. The dorsal surface of the orbitalauricular chord is attached to a slip of the levator palpebra, while the ventral surface is attached to a slip of the depressor palpebra. These attachments produce a simple mechanism for the elevation and depression of the lower earflap, and thus the opening and closing of the meatal aperture. The caudal surface of the orbitalauricular chord has connective tissue links to the rostral margin of the lower earflap. The morphology of the orbitalauricular chord appears to explain both the mechanics of the lower earflap and the functional coupling between the lower eyelid and lower earflap.

## Graphical abstract

The orbitalauricular chord is a specialized segment of dense irregular connective tissue that functionally links the lower eyelid and lower earflaps of the American alligator (Alligator mississippiensis).

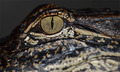

## 1 INTRODUCTION

The Crocodylomorpha arose approximately 230 mya in the late Triassac (Irmis et al., 2013). The Crocodylomorpha underwent considerable ecological radiation including multiple marine invasions, multiple transitions from a semi‐aquatic to fully terrestrial taxa, and even some bipedal forms (Kim et al., 2020; Wilberg et al., 2019). The Crocodylia, the crown group of the Crocodyliformes which includes all the extant taxa, arose in the early Cretaceous (Turner & Pritchard, 2015), and also underwent extensive ecological radiation. One of the characteristic features of the early (Novas et al., 2021) and extant (Montefeltro et al., 2016) Crocodylia is the presence of a meatal chamber on the dorsolateral surface of the skull enclosed, in part, by earflaps.

Shute and Bellairs  (1955) provided the first detailed description of the earflaps, and the associated musculature, of a crocodilian; though an earlier, incomplete, description was proffered by Killian (1890). As part of their description, Shute and Bellairs  (1955) described a “fibrous condensation” that they termed the Ypsilon. Though no formal biomechanical, or functional, model was presented, Shute and Bellairs  (1955) argued that the Ypsilon played an active role in both depressing and elevating the lower earflap, though they noted their mechanical model was incomplete. In the Crocodile chapter of Wever's treatise on the reptile ear (1978), he challenged Shute and Bellairs anatomical description of the Ypsilon and discounted their functional hypotheses. Neither the morphology nor the functional role of the Ypsilon appears to have been explored since Wever (1978).

The three eyelids of Crocodylians (hereafter termed the upper, lower, and inner eyelid) have been described extensively (e.g., Grigg & Gans, 1993; Oriá et al., 2015). So too has the asymmetry of the Crocodylian earflaps; the upper earflap is larger and more mobile caudally, while the lower earflap is larger and more mobile rostrally (Montefeltro et al., 2016; Shute and Bellairs  1955). Less attention has been paid to the coordination between these two sets of anatomically‐adjacent epithelial covers; Garrick and Saiff (1974) described a coordinated elevation of the lower eyelid and lower earflap during submergence of *Caiman sclerops*. Though not as well‐developed as in some other vertebrates (Witzmann et al., 2019), retraction of the eye has been described in *Alligator* and other Crocodylians (e.g., Fleming & Fontenor, 2014). Similar medial retraction has not been described in the earflaps of crocodylians.

The present study was undertaken to provide a more detailed anatomical description of the Ypsilon structure of the American alligator (*Alligator mississippiensis*; Daudin, 1802), and to examine the role of this structure in linking the movements of the earflaps and eyelids. For reasons detailed below, hereafter the structure dubbed “Ypsilon” by Shute and Bellairs  (1955) will be referred to as the orbitalauricular chord (Oc).

## 2 MATERIALS AND METHODS

### 2.1 Specimens

Eleven specimens of the American alligator, *Alligator mississippiensis*, were examined for this study. Three of the specimens were hatchlings (total body lengths of 24–26 cm), three were juveniles (total body lengths of 63–88 cm), two were sub‐adults (total body lengths of 156–163 cm), and three were adults (total body lengths of 237–296 cm). The specimens were either purchased commercially or obtained through the courtesy of the Louisiana Department of Wildlife and Fisheries. These specimens were all used for a study on spinal and vertebral morphometrics in *Alligator* (Greer et al., 2022); since this study involved isolating cervical vertebrae, the head of each specimen was removed intact. The heads of the hatchling and juvenile specimens were fixed in 10% neutral‐buffered formalin (nbf) for at least 48 h at 4°C; the heads of the sub‐adult specimens were frozen whole, while the heads of the adult specimens were first bisected sagittally then frozen.

### 2.2 Anatomical analyses

Frozen material was thawed overnight prior to dissection. The results of the dissection were documented using a digital camera (Nikon D3100) or a dissecting microscope (Leica M80) using the IC80HD digital camera (Leica).

The heads of the nbf‐preserved specimens were transferred to 70% ethanol for storage. Each specimen was placed in RDO Rapid Decalcifier for 24–48 h, then dehydrated through an ethanol series prior to paraffin embedding. Complete serial transverse, sagittal, and frontal sections were cut (at 10 μm) through the region of the orbitalauricular chord. Sections were stained with Hematoxylin and Eosin, Masson's trichrome stain, Phosphotungstic Acid Hematoxylin (PTAH) stain, and Picrosirius Red. Microscopic anatomy was documented using a DM 4000B microscope (Leica Microsystems Inc.).

The head of one of the juvenile specimens was pre‐treated in Lugol's solution and then scanned at the University of Texas High‐Resolution X‐ray CT Facility using a custom‐built North Star Imaging scanner in volume mode. The X‐ray source was set to 130 kV and 0.14 mA with an aluminum foil pre‐filter. A total of 3600 projections were acquired over 360 degrees of rotation, at 1 frame per second with no frame averaging and no detector binning. A beam‐hardening correction of 0.25 was applied; the resulting slices measured 1979x1979 pixels and had a voxel resolution of 9.65 microns. The resulting DICOM images were examined, and a 3‐D reconstruction was performed, using Dragonfly (Object Research Systems).

## 3 RESULTS

### 3.1 Superficial and skeletal morphology

**FIGURE 1:**
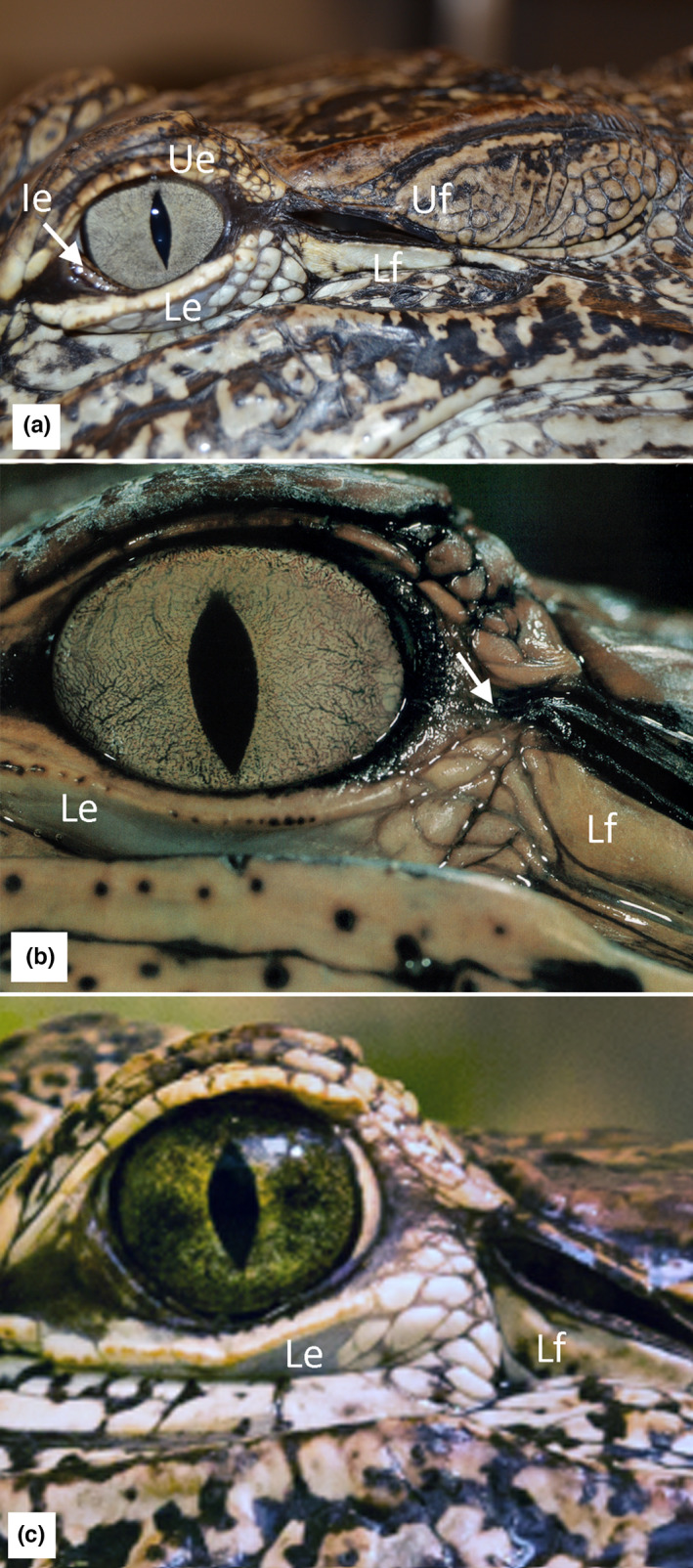
Superficial morphology of *Aligator mississippiensis*. (a) sub‐adult (143 cm total body length); (b) hatchling (21 cm total body length) showing the close apposition of the orbit and meatal aperture (arrow); (c) sub‐adult (159 cm total length); the distance between the orbit and meatal aperture has increased, and the bare epithelium has been covered with small granular scales. Ie, inner eyelid; Le, lower eyelid; Lf, lower earflap; Ue, upper eyelid; Uf, upper earflap.

*Alligator mississippiensis* has three mobile eyelids; an upper eyelid (which includes a bony plate in larger individuals), a lower eyelid, and an internal eyelid (nictitating membrane) which retracts into the rostral‐ventral portion of the orbit (Figure 1). The upper earflap is the largest of the two. It covers all of the meatal chambers caudally and then narrows rapidly near the rostral margin of the meatal chamber (Figure 1); dorsal rotation of the upper earflap exposes the majority of the meatal chamber and tympanic membrane. The lower earflap is the smaller of the two meatal coverings. Caudally it is little more than an epithelial ribbon, but it expands dorsally near the rostral margin of the meatal chamber (Figure 1).

In hatchling *A. mississippiensis*, the rostral opening of the meatal chamber abuts the caudal soft tissue of the orbit (Figure 1b). The epithelium between the two is generally smooth and free of scales/scutes. Differential growth in the skull separates the orbital and meatal opening; the epithelium in between is initially smooth but with the increasing size, it is covered by small granular scales (Figure 1c). With larger individual *A. mississippiensis*, or even with preserved specimens, the orbitalauricular chord can be readily palpated at the granular scales just rostral to the meatal opening.

**FIGURE 2:**
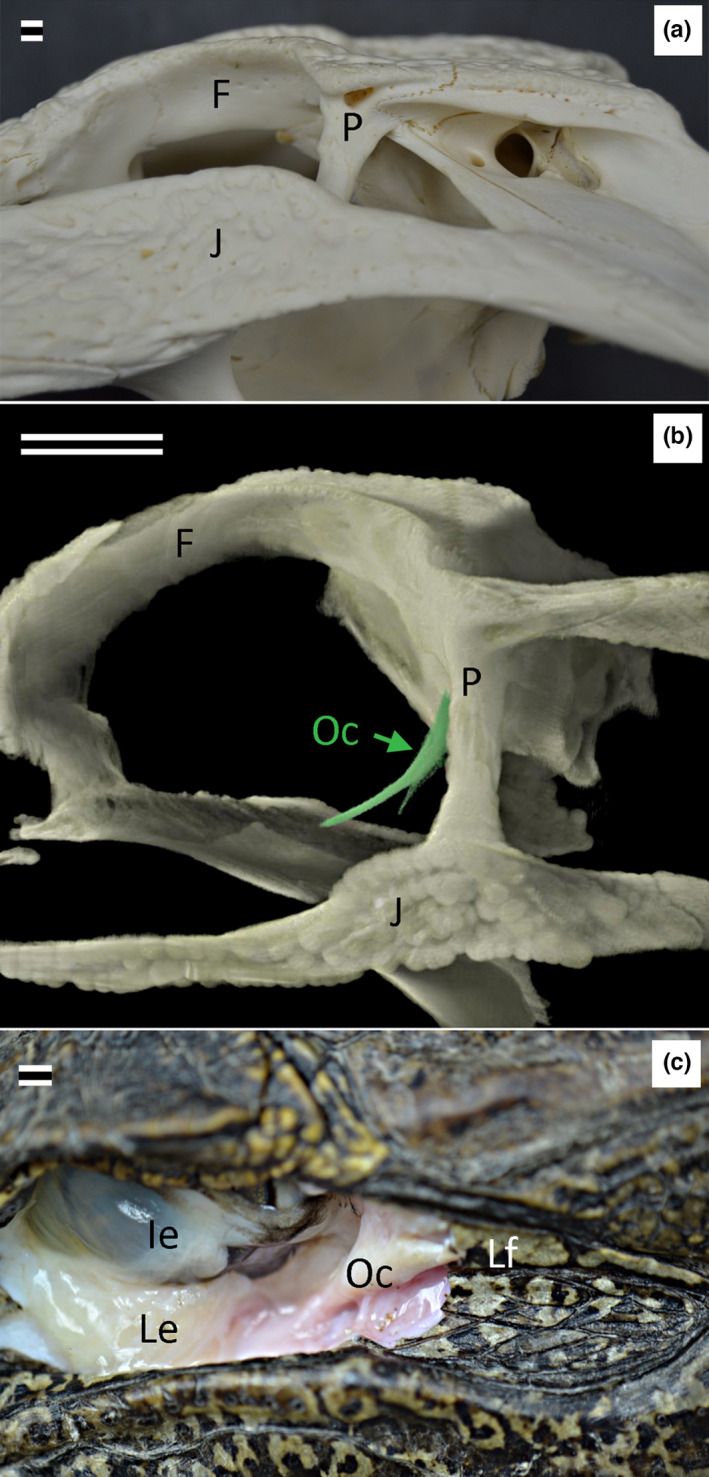
Position of the orbitalauricular chord in *Alligator mississippiensis*. (a) lateral view of the skull of a sub‐adult (172 cm total body length) specimen, the postorbital serves as an osseous boundary between the rostral orbit and the caudal meatal chamber; (b) 3‐D reconstruction (based on micro‐CT data) showing the orbitalauricular chord relative to the bony landmarks; (c) superficial dissection of an adult (237 cm total body length) specimen; to increase the exposure the dorsal portion of the jugal has been removed. Note the similarity in the course and orientation of the orbitalauricular chord in (b) and (c). Scale bars in all images are 5 mm. F, frontal; Ie, inner eyelid; J, jugal; Le, lower eyelid; Lf, lower earflap; Oc, orbitalauricular chord; P, postorbital.

The orbital cavity of *A. mississippiensis* is bordered caudally by the ventral (and roughly horizontal) jugal bone, the dorsal (and roughly horizontal) frontal bone, and the nearly vertical postorbital bone that fuses to the frontal and jugal (Figure 2a). The Oc is located immediately rostrolateral to the postorbital; it does not extend dorsally as far as the frontal but does extend ventrally beyond the dorsal surface of the jugal (Figure 2b). In non‐preserved specimens, the Oc can be found superficially immediately caudal to the orbit (or rostral to the meatal aperture); it is characterized by a solid texture and a bright white coloration. The Oc is oriented obliquely, with the ventral portion located more rostral and lateral than the dorsal portion (Figure 2c).

### 3.2 Morphology and histological composition

**FIGURE 3:**
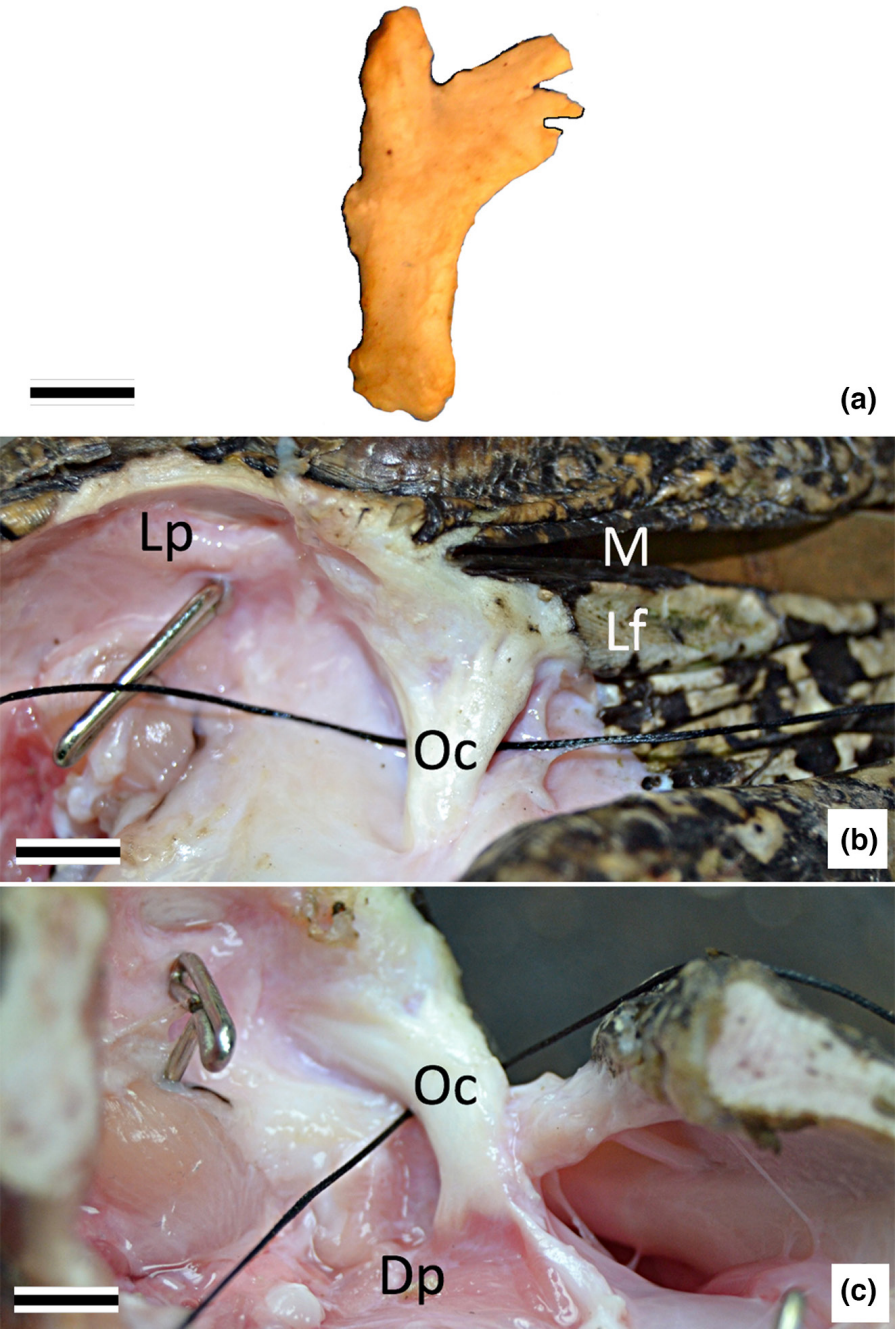
Morphology of the orbitalauricular chord in *Alligator mississippiensis*. (a) photograph of the lateral surface of the oc excised from a sub‐adult (163 cm total body length) specimen. (b) lateral view of a dissection of the oc from an adult (248 cm total body length) specimen; the suture has been passed between the oc and the postorbital process. (c) rostral view of the same dissection, the eye has been removed to enhance the visibility of the oc. Scale bars are 5.0 mm. Dp, depressor palpebra; Lp, levator palpebra; Oc, orbitalauricular chord.

The Oc is a relatively straight, non‐splitting structure, which shifts from a rostrolateral to a rostral position (relative to the postorbital process) as it courses ventrally. The dorsal portion of the Oc is nearly stellate (Figure 3a,b); a caudal group of fibers integrates into the lower earflap just ventral to the rostral margin of the meatal aperture, while a (smaller) rostral group of fibers extend slightly further dorsally and integrate into the musculoskeletal elements of the caudal margin of the orbit. Ventrally the Oc ends “abruptly,” rather than tapering. The ventral end of the Oc is embedded in muscle; there are short fibers extending both medially and laterally, but the Oc itself does not branch or split (Figure 3a,c).

**FIGURE 4:**
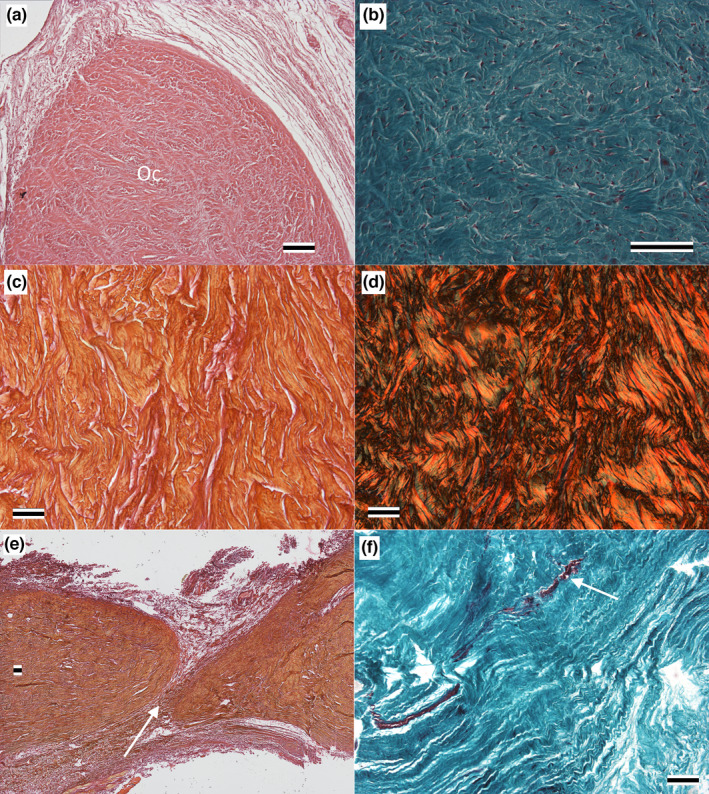
Histological features of the orbitalauricular chord of *Alligator mississippiensis*. (a) transverse section, hematoxylin, and eosin‐stained, showing the variation of loose connective tissue surrounding the orbitalauricular chord and the lack of a synovial layer around the chord. (b) frontal section, Masson's trichrome stain, indicating the dense irregular composition of the orbitalauricular chord. (c and d) same frontal section, stained with picrosirius red, imaged with brightfield (c) and polarized light (d), demonstrating the marked variation in collagen fiber orientation, (e) sagittal section, picrosiurius red stain, loose connective tissue plate or seam (arrow) running at least partially through the orbitalauricular chord. f) frontal section, Masson's trichrome, though not abundant, neurovascular elements were found within the orbitalauricular chord (arrow). Scale bars are all 100 μm.

The Oc is composed of dense irregular connective tissue. The surface of the Oc can be indistinct at times, given the integration with surrounding tissue (see below and Figure 4a); no evidence of a synovial or mucosal sheath was observed. Collagen fibers formed the vast majority of the Oc, and these fibers showed no clear pattern of orientation (Figure 4b–d). There were some short septa or planes of loose connective tissue that projected into the Oc (Figure 4e); these loose connective tissue planes never passed completely through or partitioned the Oc. Though sparse, there were neurovascular bundles within the Oc (Figure 4f); no chondrocytes were ever observed.

**FIGURE 5:**
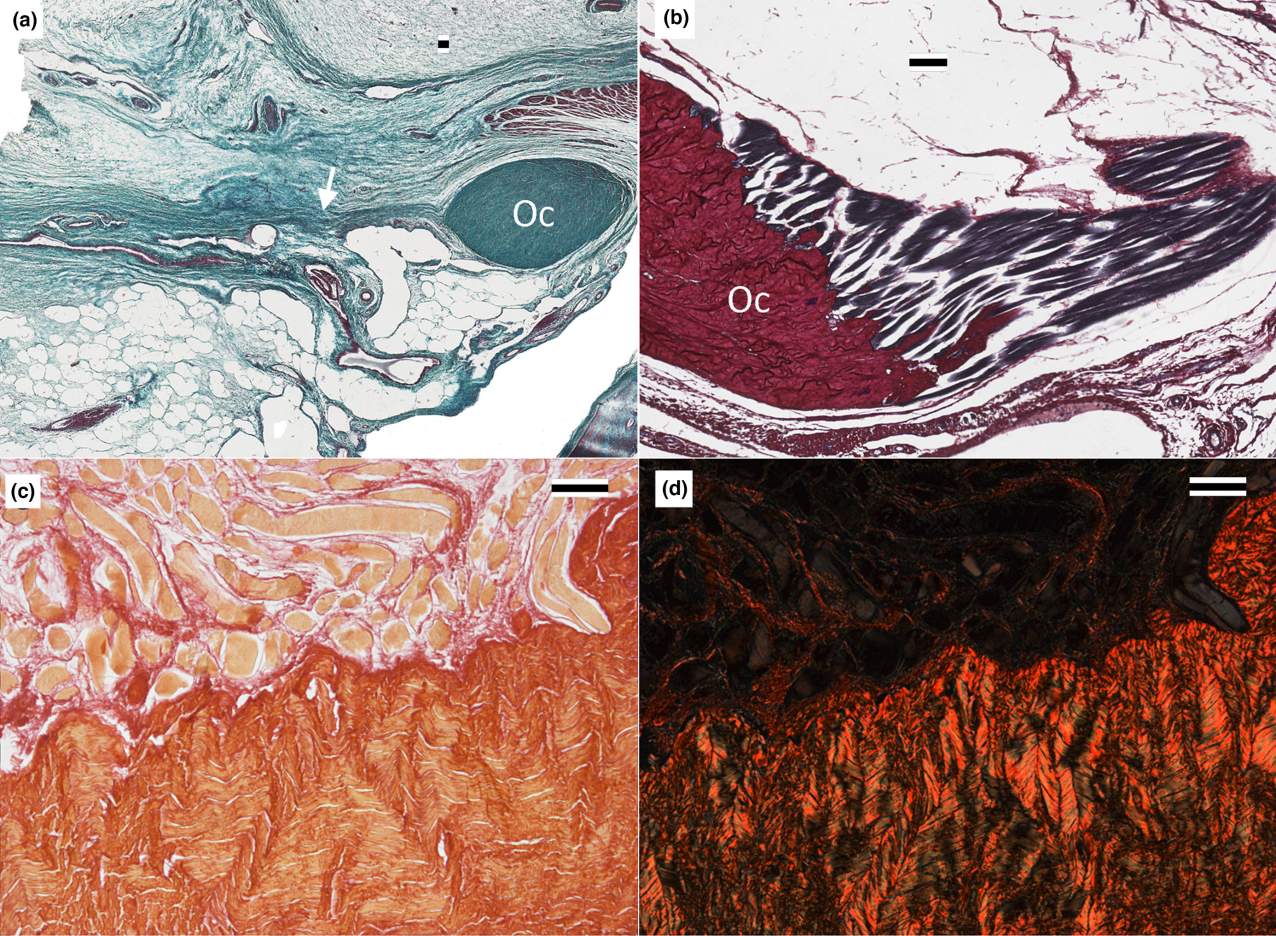
Histological attachments on the orbitalauricular chord of *Alligator mississippiensis*. (a) frontal section, Masson's trichrome stain, showing a band of dense regular connective tissue (arrow) attaching to the orbitalauricular chord. (b) frontal section, PTAH stain, showing skeletal muscle fibers inserting onto the orbitalauricular chord. (c and d) same sagittal section, stained with picrosirius red, imaged with brightfield (c) and polarized light (d), note that the collagen fibers are not aligned with the skeletal muscle fibers. Scale bars are all 100 μm. Oc, orbitalauricular chord.

Along the length of the Oc, there were three forms of contact or attachment observed (the specific attachments are detailed below). The surrounding loose connective tissue frequently abutted the surface of the Oc in a way that suggested possible adherence (Figure 4a). Larger amalgams of dense regular connective tissue are simply integrated with the collagen fibers of the Oc (Figure 5a). Skeletal muscle fibers are inserted onto the surface of the Oc (Figure 5b). Using picrosirius red stain to document the orientation of the collagen fibers (Figure 5c,d) revealed that even at the local site of muscle fiber attachment the collagen fibers exhibited an essentially “random” arrangement.

### 3.3 Spatial position of the orbitalauricular chord

**FIGURE 6:**
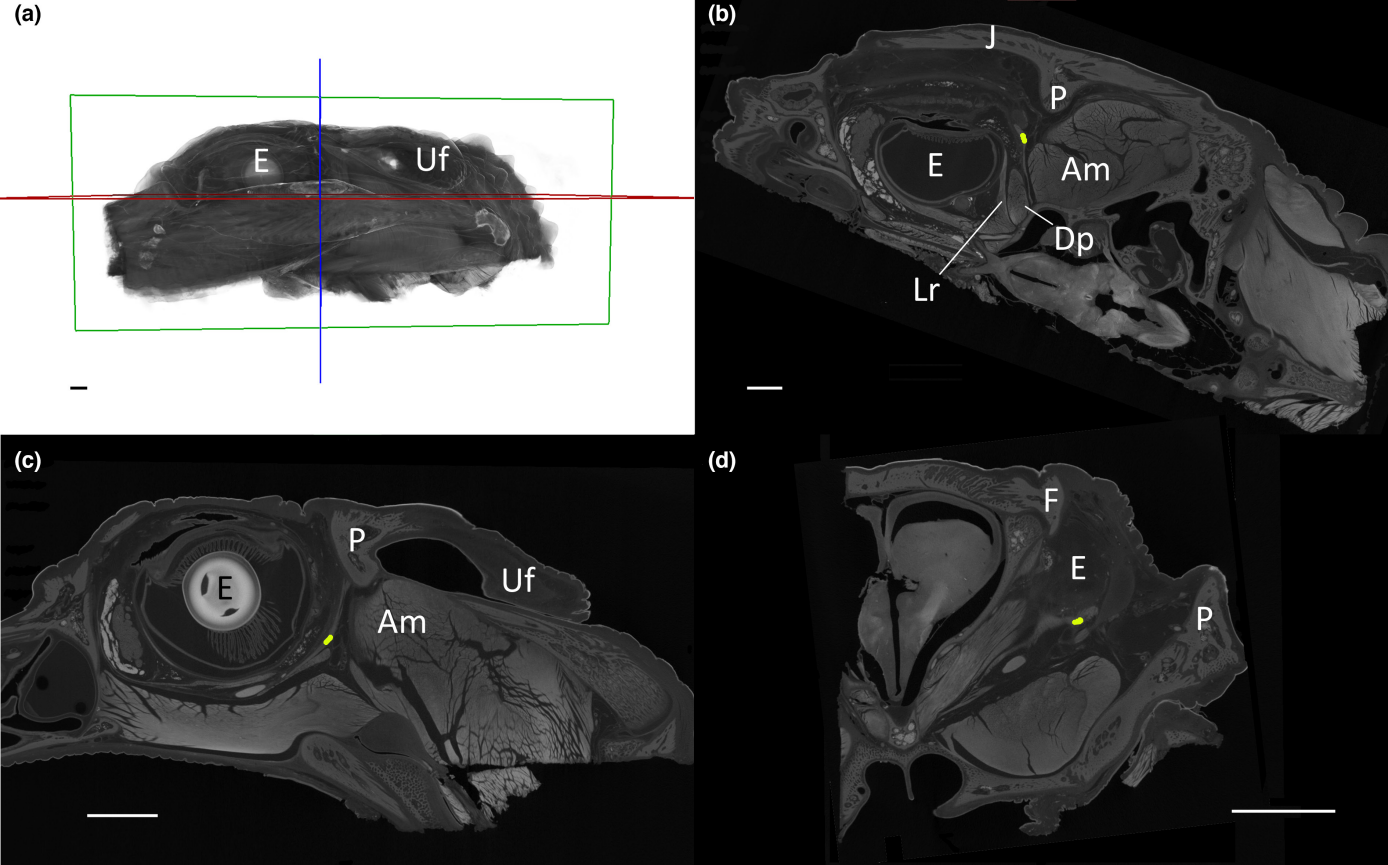
Anatomical position of the orbitalauricular chord. (a) 3‐D reconstruction of the sample used for the micro‐CT imaging; the planes for the other three images are color‐coded. Images from the frontal (b), sagittal (c), and transverse (d) planes; the point of intersection of the three planes falls on the orbitalauricular chord and is highlighted in yellow. Scale bars are all 5 mm. Am, adductor mandibulae externus superficialis; Dp, Depressor palpebra; E, eye; F, frontal; J, jugal; Lr, lateral rectus; P, postorbital; Uf, upper earflap.

The orbitalauricular chord is located in an anatomical space defined rostrally by the caudal and inferior surfaces of the eye, and caudally by the postorbital bone and the rostral surface of the jaw adductors (Figure 6). The ventral portion of the Oc is medial to the jugal, while the dorsal portion is covered by the scalation present between the orbit and the auditory meatus (Figure 6). The Oc is oriented obliquely with the ventral portion more rostral and lateral than the dorsal portion; the oblique position of the Oc means that it is best seen in the sagittal (Figure 6c) and transverse (Figure 6d) planes.

### 3.4 Attachments of the orbitalauricular chord

**FIGURE 7:**
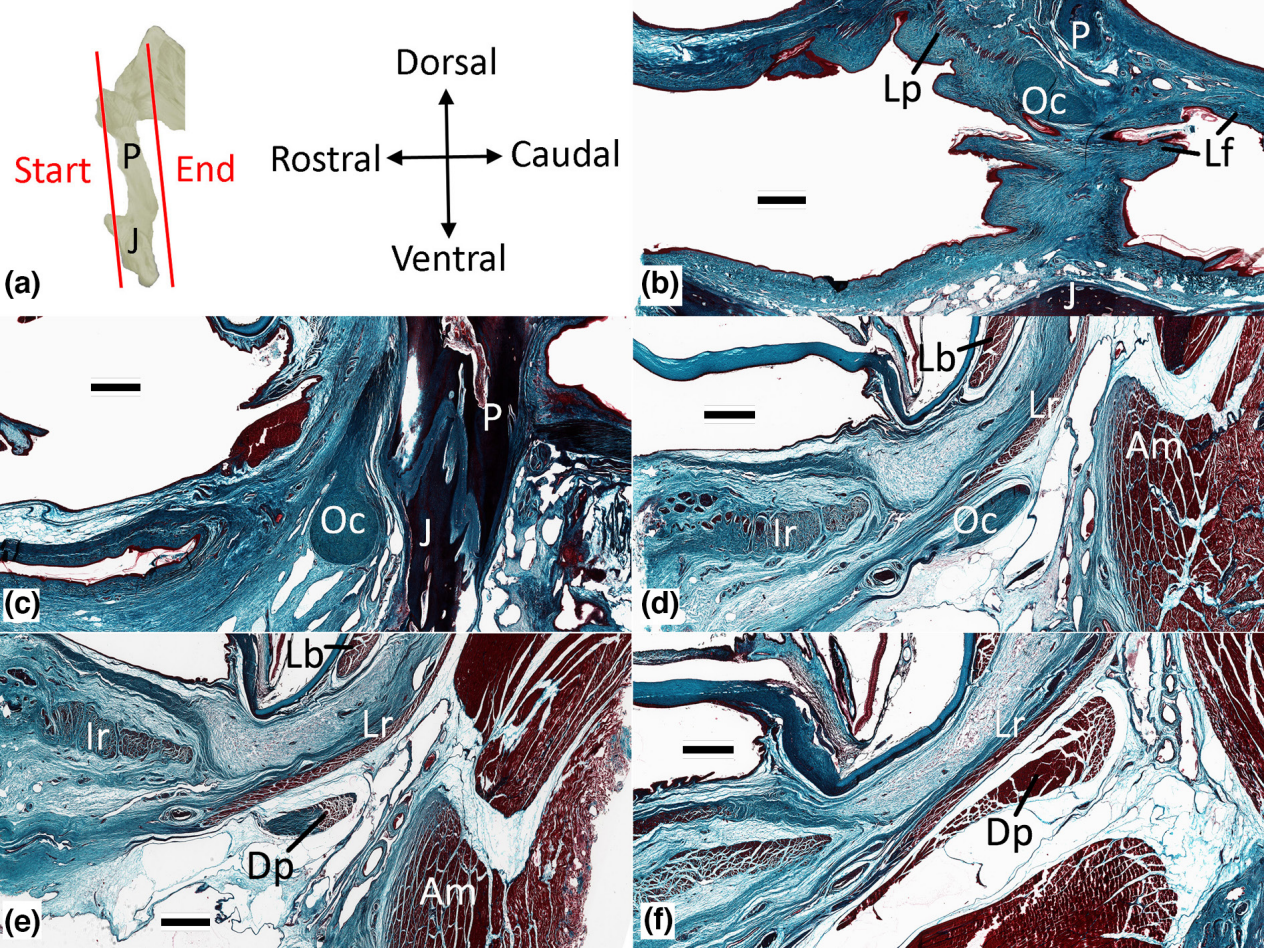
Histological sections through the oribtalauricular chord. a) sequence of sections relative to the jugal (J) and postorbital process (P); each parasagittal section (b‐f) is oriented as shown. Scale bars are all 1 mm. Abbreviations: Am, adductor mandibulae externus superficialis; Dp, Depressor palpebra; Ir, inferior rectus; J, jugal; Lb, levator bulbi; Lf, lower earflap; Lp, levator palpebra; Lr, lateral rectus; Oc, orbitalauricular chord; P, postorbital.

**FIGURE 8:**
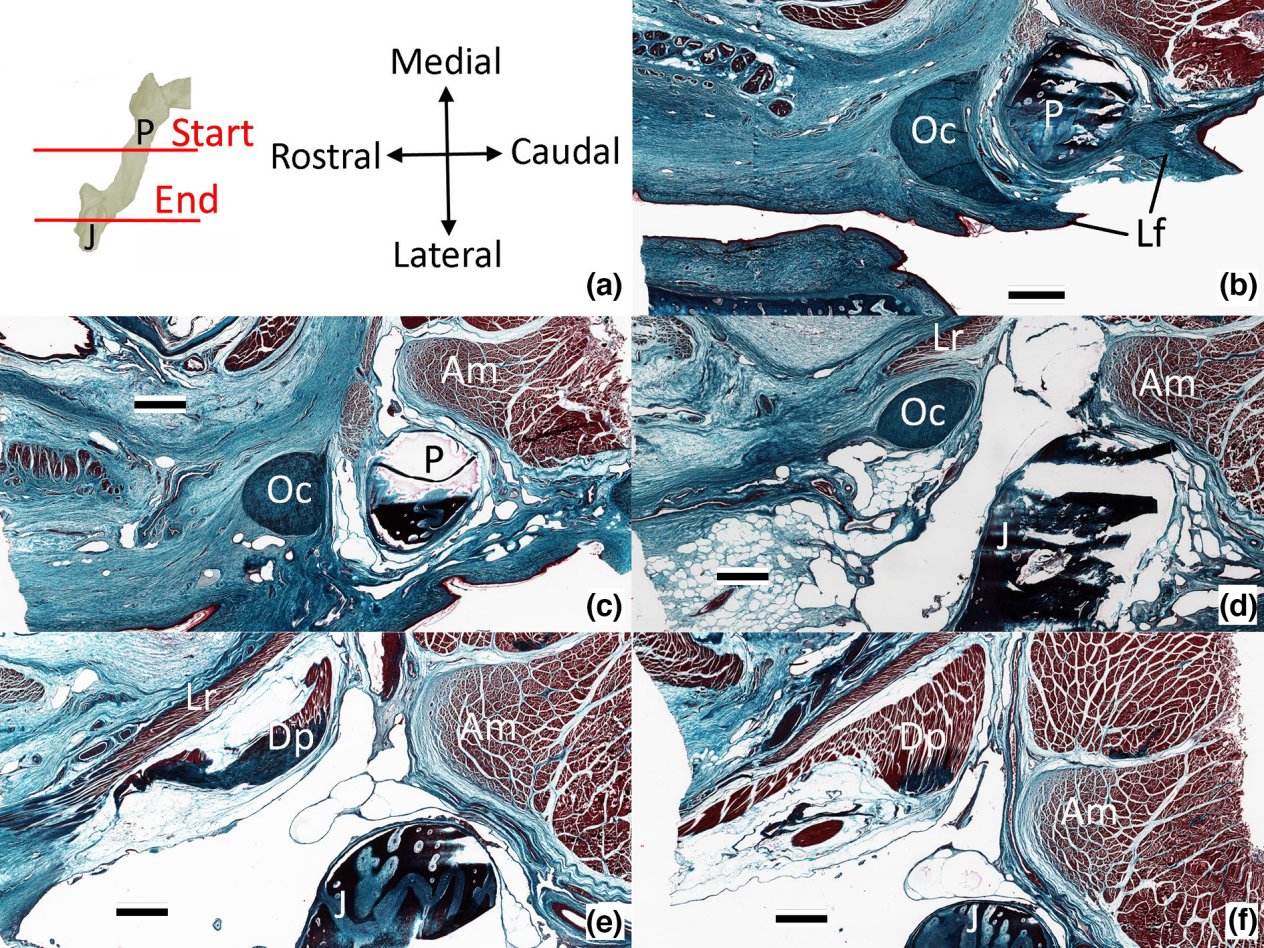
Histological sections through the oribtalauricular chord. (a) sequence of sections relative to the jugal (J) and postorbital process (P); each frontal section (b–f) is oriented as shown. Scale bars are all 1 mm. Abbreviations: Am, adductor mandibulae externus superficialis; Dp, Depressor palpebra; J, jugal; Lf, lower earflap; Lr, lateral rectus; Oc orbitalauricular chord; P, postorbital.

Dorsally the Oc has two well‐developed attachments. The dorsocaudal surface of the Oc is bound to the rostral margin of the lower earflap (Figures 3b, 7b, 8), while the dorsorostral surface is bound (more indirectly) to the connective tissue of the lower eyelid and serves as an insertion site for the levator palpebra. The Oc is always in proximity to the postorbital process, but there is little direct contact between the two elements; neurovascular elements and loose connective tissue are found between the Oc and the postorbital process (Figures 7 and 8). The one exception is a band of dense regular connective tissue (histologically distinct from the Oc) that extends dorsally from the Oc to attach to the ventral surface of the postorbital (Figure 7c). As the Oc extends ventrally of the postorbital process, there is a second (smaller) connective tissue attachment; these collagen fibers extend rostrolaterally from the Oc to reach the epithelium and deep connective tissue of the lower eyelid (Figure 8d). The ventral tip of the Oc serves as an insertion site for a slip of the depressor palpebra (Figure 7d,e and Figure 8d,e).

### 3.5 Functional hypothesis

**FIGURE 9:**
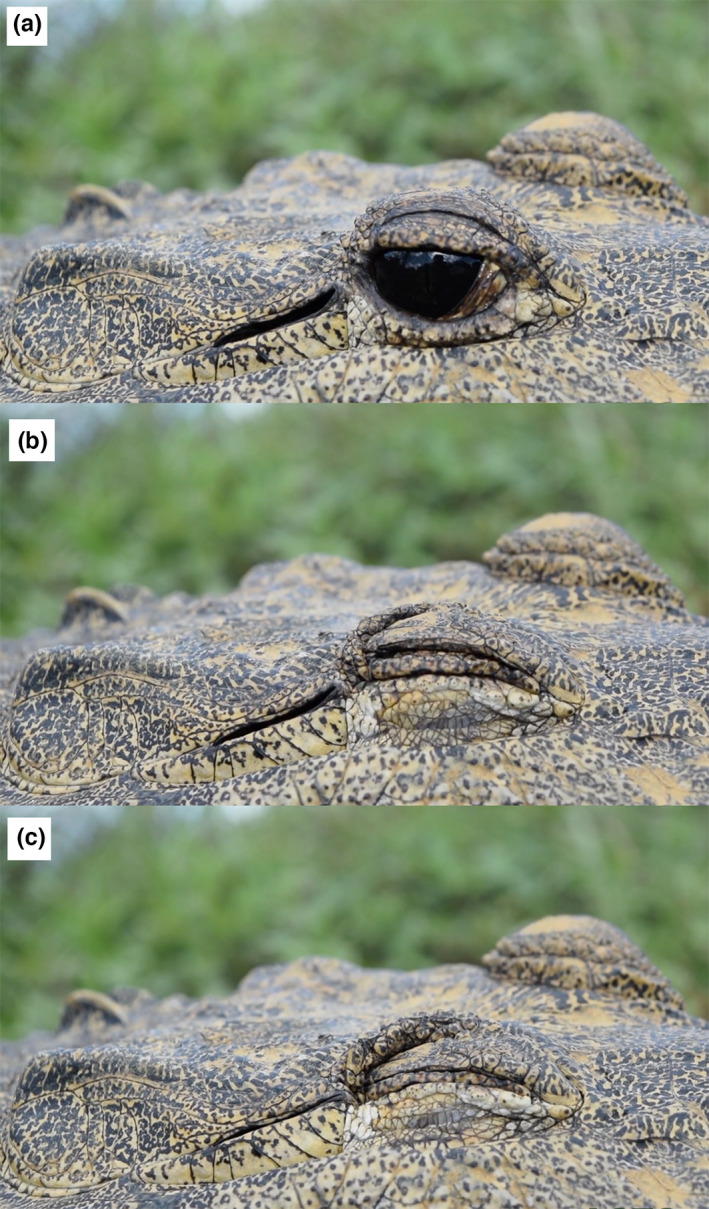
Images from a video sequence (kindly provided by the American Crocodile Education Sanctuary) of an American crocodile (*Crododylus acutus*) reflexively elevating the lower earflaps and lower eylids (compare frames a and b), then retracting the eye (compare frames b and c).

In *Alligator mississippiensis*, and perhaps all crocodylians, the reflex elevation of the lower eyelid is linked with the elevation of the lower earflap (Figure 9a,b). Often there is a “second stage” to this process, in which there is retraction of the eye within the orbit. Retraction of the eye causes deformation of the epithelium/scales linking the caudal edge of the orbit and the rostral margin of the meatal aperture (Figure 9c). No retraction of the earflaps was observed in *A. mississippiensis*.

**FIGURE 10:**
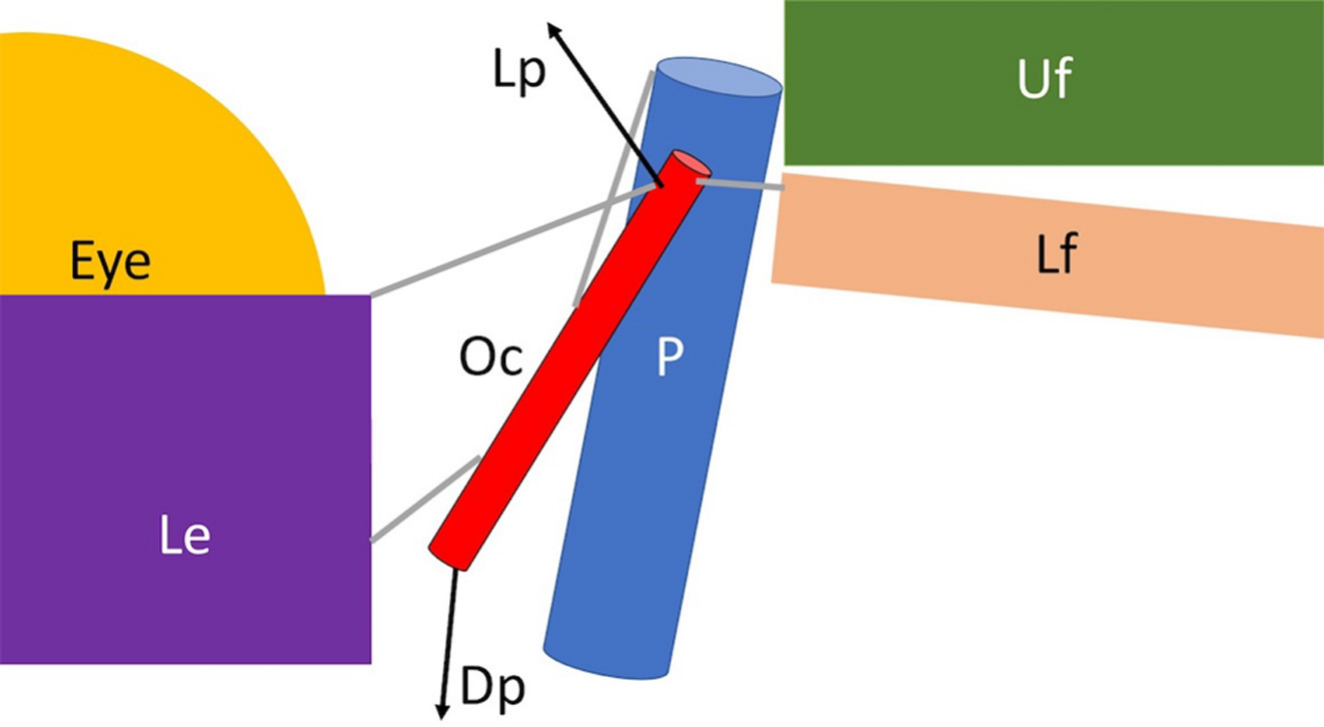
Simple functional model of the orbitalauricular chord. The oc is directly bound to the lower earflap and lower eyelid, but is mobile relative to the postorbital bone and process. Contraction of the levator palpebra elevates the oc and thus the lower earflap and lower eyelid, while contraction of the depressor palpebra results in the opposing depression. The connective tissue attachments of the oc are indicated by gray lines. Dp, depressor palpebra; Le, lower eyelid; Lf, lower earflap; LP, levator palpebra; Oc orbitalauricular chord; P, postorbital process; Uf, upper earflap.

The Oc physically links the lower earflap to the lower eyelid (Figure 10). The Oc is free of direct attachment to the postorbital process (with the exception of the dorsally coursing band of connective tissue). Contraction of the portion of the levator palpebra that inserts on the dorsal end of the Oc should result in linked elevation of the lower earflap and lower eyelid. Similarly, contraction of the portion of the depressor palpebra that inserts on the ventral end of the Oc would result in a linked depression of the lower earflap and lower eyelid (Figure 10). The ventral displacement of the Oc would be checked by the connective tissue band anchoring the Oc to the postorbital. In a fresh specimen, manual elevation and depression of the Oc are adequate to produce linked movements of the lower eyelid and lower earflap (see Videos S1 and S2).

Contraction of the retractor bulbi causes the eye to move medially and ventrally in the orbit, this likely causes a slight medial displacement/rotation of the oc which is what produces the slight depression of the epithelium between the eye and meatal aperture (Figure 9c).

## 4 DISCUSSION

Shute and Bellairs  (1955) described the dorsal end of the orbitalauricular chord (Oc), which they dubbed the Ypsilon, as dividing into a medial and lateral branch. The present study examined more specimens of *Alligator mississippiensis*, over a broader size range, using more techniques, and could not support the “Ypsilon” description. The dorsal end of the Oc is almost stellate (Figure 3) from an abundance of connective tissue attachments. One group of attachments extends caudally to integrate into the rostral margin of the lower earflap; these connective tissue attachments are histologically distinct from the Oc itself. The other group of attachments on the dorsal surface of the Oc are with connective tissue and muscle fibers; these other elements course from the Oc in the rostral and dorsal directions, and, like the attachments on the caudal surface, are histologically distinct from the Oc itself. The findings of the present study agree with those of Shute and Bellairs  (1955) in that the ventral end of the Oc terminates with a muscle attachment, which we both identify as a portion of the depressor palpebra.

Wever (1978) found, as the present study did, that the “medial arm” described by Shute and Bellairs  (1955) was not evident. Furthermore, the mechanical model Shute and Bellairs  (1955) described for the lower earflap was dependent on a fixed anchor between the dorsal end of the Oc and the postorbital. Wever (1978) found the Oc to be mobile relative to the postorbital, which was clearly the case with the material examined for the present study (see Videos S1 and S2).

Shute and Bellairs  (1955) postulated that elevation of the lower earflap could be achieved by smooth muscle coursing around the meatal aperture in a sphincter‐like arrangement. In contrast, the present study found a clear connection between the levator palpebra and the Oc. Wever (1978) argued that the levator bulbi muscle could function (indirectly) to elevate the Oc. Neither Shute and Bellairs  (1955) nor Wever (1978) directly addressed the mechanical linkage between the lower earflap and the lower eyelid. The present study identified connective tissue attachments between the Oc and both the lower eyelid and the lower earflap; furthermore, the present study documented specialized portions of two orbital muscles (levator palpebra and depressor palpebra) which insert on the Oc and provide obvious opportunities for shared neural control. The combined functional influence of the Oc can be demonstrated by manual displacement of the Oc in a fresh (non‐preserved) specimen (see Videos S1 and S2). Garrick and Saiff (1974) questioned the anatomical findings and biomechanical model of Shute and Bellairs  (1955), and noted that they were preparing an independent anatomical analysis to explain the coupled closing of the ears and eyes; this planned anatomical analysis does not appear to have been published.

The present study used the term “chord” to refer to this mass of dense irregular connective tissue. The term “ligament” seems a poor choice since these collagen fibers neither interconnect two bones, span a joint, nor show a common orientation (Figures 4 and 5). A stronger case could be made for considering the Oc a tendon, particularly a tendon like that of the omohyoid (Rai et al., 2008) in which there are two muscle bellies separated by a central tendon. In the case of the Oc, it would be a linking tendon between the levator palpebra dorsally and the depressor palpebra ventrally. The histological structure of the Oc precludes recognizing it as a tendon, particularly how the collagen fibers are nearly perpendicular to the long axis of the muscle fibers (Figure 5). The term “chord” was used in the present contribution since it was not associated with any specific histological organization.

None of the sections cut through the Oc, whether from hatchling or adult specimens, had chondrocytes or any evidence of chondrification or ossification. Most of the connections with the Oc involved dense regular connective tissue; essentially the same tissue and tissue density, just a different organization. This proved to be problematic in that while the micro‐CT images generated as part of this study could distinguish the Oc from the adjacent postorbital process, they were not adequate to resolve the precise borders of the Oc (see Figure 6); that is why only the central portion of the Oc was reconstructed in Figure 2.

Though not the focus of the present contribution, it is interesting to speculate on the evolutionary origin of the Oc. The meatal chamber, and the associated earflaps, appear to be a regular feature of Crocodyliforms (Montefeltro et al., 2016). The larger upper earflap is controlled by a series of muscles, mainly on the caudal margin, which is derived from the lateral jaw muscles (e.g., Edgeworth, 1935). A finer seal to the meatal aperture is gained by having both upper and lower, independently mobile, earflaps. The musculature responsible for displacing the lower earflap is derived not from the lateral jaw muscles, but rather from the orbital musculature. The recent 3‐D atlas of the cranial nerves and associated muscles of *A. mississippiensis* (Lessner & Holliday, 2020) is a valuable resource for exploring how the orbital muscles could have specialized to drive the lower earflap. If a slip of the depressor palpebra developed a tendinous extension that reached the lower earflap, it would resemble something similar to the majority of the Oc and the caudal portion of the Oc's stellate proximal end. If the levator palpebra developed a tendinous extension that fused to the Oc, it would produce the rostral portion of the Oc's stellate dorsal end. If these two tendons coursed parallel and fused, it could account for the diversity of collagen fiber orientation (Figure 4) as well as the presence of clefts within the Oc (Figure 4e). This hypothesis for the origin of the Oc is consistent with the simpler description offered by Shute and Bellairs  (1955) that the Oc is a “fibrous condensation,” and is compatible with other connective tissue specializations known in the crocodylian head (e.g., Shimada et al., 1993; Tsai & Holliday, 2011) The hypothesis presented herein for the evolution of the Oc could be explored through a detailed developmental study.

## AUTHOR CONTRIBUTIONS

BAY designed this study and drafted the original version of the manuscript. All of the authors participated in the anatomical analysis and assisted in the editing/preparation of the final manuscript.

## CONFLICT OF INTEREST

The authors declare that they have no conflicts of interest.

## Supporting information


Video S1

Video S2

## Data Availability

The micro‐CT files produced during this study have been archived on Morphospace (doi); the histological slides generated during this study are available from the corresponding author.
